# Cognitive and motor improvement by tummy time practice in preemies from low-income settings: a randomized clinical trial

**DOI:** 10.3389/fpsyg.2024.1289446

**Published:** 2024-09-18

**Authors:** Sabrinne Suelen Santos Sampaio, Nathalia Allana Amorim Rodrigues, Thalyson Luiz Gomes Souza, Julia Raffin Moura, Ingrid Guerra Azevedo, Carolina Daniel Lima-Alvarez, Silvana Alves Pereira

**Affiliations:** ^1^Department of Physical Therapy, Federal University of Rio Grande do Norte, Natal, Brazil; ^2^Graduation Program in Sciences of Rehabilitation, University of Brasilia (UNB), Brasília, Distrito Federal, Brazil; ^3^Research Directory, Catholic University of Temuco, Temuco, Chile

**Keywords:** motor skill, premature birth, tummy time, prone position, infant development, low-income countries, intervention

## Abstract

**Introduction:**

Early intervention and parental education for preemies are limited in some low-income countries. Thus, this study aimed to assess whether daily tummy time (TT) associated with usual care (UC) enhances motor and cognitive development in preemies from low-income countries. The main and secondary aim was to assess prone head elevation (PHE) and motor and cognitive functions, respectively.

**Methods:**

Thirty-one preemies with a mean gestational age at birth of 33.3 ± 1.6 weeks and weighing <2,500 g were included and 18 completed all assessments. Parents from the TT group were asked to perform TT with their preemies for at least 20 min during daily activities and playtime. Motor and cognitive functions were assessed by a blinded examiner using the motor and cognitive composite scores of Bayley-III after two and four months of corrected age. PHE was given by the angle from the tragus of the ear to the supporting surface of the infant; measurements were obtained using the Kinovea® software at baseline, after two, three, and four months of corrected age.

**Results:**

The Bayley-III motor composite score of the TT group was higher than the UC group after two and four months of corrected age. The PHE angle increased over time but did not differ between groups.

**Discussion:**

Nevertheless, TT expanded the perspective of preemies to explore their bodies and environment, favoring the stimuli for motor and cognitive patterns. The loss of participants (31%) was one of the limitations of the study, illustrating the challenge of providing continued early interventions to preemies from low-income countries. In this sense, TT practice is a home intervention that may improve motor and cognitive function of preemies immediately after hospital discharge.

## Introduction

Tummy time (TT) is a crucial intervention that enhances motor skills in infants (World Health Organization, 2023). TT was formerly recommended to combat musculoskeletal complications from the campaign Safe to Sleep and involves placing the infant in the prone position for some time under the supervision of an adult (World Health Organization, 2019). Infants during TT are exposed to new experiences that develop motor, cognitive, and sensory abilities needed to explore and perceive the environment and their bodies. Thus, TT may enhance the development of infants, especially in the first months (Adolph and Hoch, 2019). In this way, new experiences provide a wide primary and secondary variability in the development (Hadders-Algra, 2018), which may be useful for developing problem-solving expertise. For instance, the infant is encouraged to lift the head, strengthening several muscular groups (e.g., cervical extensor, paravertebral, and scapular stabilizing muscles) and increasing cervical mobility. Moreover, the weight-bearing experience in the arms favors the development of manual and manipulative skills since it improves mobility and strengthens the muscles of the arms. Thus, TT develops muscle synergy needed for movement control and the acquisition of new abilities. In addition, the required visual adjustments improve the visual function, enhancing visual accuracy, focus, and attention (Adolph and Hoch, 2019; Kretch et al., 2014; Lee and Galloway, 2012; Soska and Adolph, 2014). In this sense, Kretch et al. (2014) presented that the changed visual experience may lead infants to have divergent experiences and different opportunities for learning. In their study, 13 infants experienced different postures wearing a head-mounted eye-tracker recording gaze direction and head-centered field of view.

Besides the biomechanical and functional changes, the new visual fields develop new spatial and movement concepts, as well as cause-effect associations. Consequently, preemies are exposed to new learning experiences and exploration of the environment. Hence, improved perception and movement planning increase movement possibilities based on decision-making and problem-solving approaches, refining cognitive abilities (Adolph and Hoch, 2019). In this sense, TT is an important intervention that improves the neurodevelopment of preemies and is highly recommended for infants up to six months of age by World Health Organization (2019). Additionally, TT fosters bonding between baby and parents and does not require special equipment, representing an accessible and feasible intervention for daily life. Thus, TT may be effective as a preterm follow-up intervention, especially in low-income settings, in which many families present difficulties in health assessment due to socioeconomic or transportation problems (Sampaio et al., 2023).

Brazil is a low-income country that presents a large socioeconomic diversity (Brazil, 2024), and about 70% of the neonatal care services and early intervention programs are concentrated in metropolitan areas (Ministério da Saúde, 2024). Moreover, most health services for outpatient follow-up of preemies currently use the watchful waiting approach, which only refers the preemie to an early intervention service when a neurodevelopment delay is detected (Lyne et al., 2022). The Brazilian northeast is the second poorest Brazilian region, characterized by low educational attainment and poor social support. Thus, preemies with neurodevelopmental delays from this region are not referred for early intervention service. Therefore, the literature shows that the interventions during this critical developmental window are inadequate due to the lack of knowledge of what, how, or when to stimulate a preemie (Baggett et al., 2020; Novak et al., 2020; Rosenberg et al., 2013; Twardzik et al., 2017; Watson, 2013). Thus, challenges in the current practice hindered the follow-up of infants with high risk for developmental delays (Lyne et al., 2022). Furthermore, the specialized care followed by caregivers is affected by barriers, such as difficulties in transport access, poor social support, limited time at home, and medical concerns regarding the mother or the preemie (Smith et al., 2017). The impact of this issue is enhanced after hospital discharge, as caregivers are challenged to keep the same level of care provided by the multiprofessional team while coping with anxiety, isolation, and financial constraints.

Although TT is important for the neurodevelopment during early childhood (Boutot and DiGangi, 2018; Clark and Kingsley, 2020; Hewitt et al., 2017; World Health Organization, 2019), literature still lacks evidence regarding its effects on motor and cognitive functions in preemies. Thus, this study aimed to verify whether TT improve motor and cognitive development of preterm infants, assessing prone head elevation (PHE), and motor and cognitive functions after TT.

## Materials and methods

### Study setting and participants

This single-blinded, randomized clinical trial was conducted at the Januario Cicco maternity School of the Federal University of Rio Grande do Norte (Natal, Rio Grande do Norte, Brazil) and was approved by the research ethics committee of the Federal University of Rio Grande do Norte (CAAE: 44712221.5.0000.5537). This study was registered in the Brazilian Registry of Clinical Trials (REBEC; RBR-2nwkr47), and the protocol was previously reported (Sampaio et al., 2023). Written informed consent and the authorization of the image use were obtained from the caregivers of the preemies on behalf of both before the start of the study.

### Participants and recruitment

The families were recruited at the Januario Cicco pediatrics and childcare ambulatory care maternity school 48 h after hospital discharge (corresponding to the first outpatient consultation); recruitment occurred on spontaneous demand. Table 1 summarizes the characteristics of the participants.

**Table 1:** Description of families and infants.

	Tummy time (*n* = 16)	Usual care (*n* = 15)	*p*-value
Families			
Maternal age^*^	28 (5.96)	30 (8.25)	0.39
Number of children^**^	2 (1–4)	2 (1–4)	0.96
Marital status (*n*/%)^∞^			1.00
*Single*	3 (18.80)	3 (20.00)	
*Married*	13 (81.30)	12 (80.00)	
Maternal education (*n*/%)^∞^			0.27
*Elementary or less*	8 (50.00)	4 (26.70)	
*High school or more*	8 (50.00)	11 (73.30)	
Employment (*n*/%)^∞^			0.68
*Yes*	3 (18.80)	4 (26.70)	
*No*	13 (81.30)	11 (73.30)	
Residence (*n*/%)^∞^			1.00
*Metropolitan area*	7 (43.80)	7 (46.70)	
*Countryside*	9 (56.30)	8 (53.30)	
Infants			
Birth weight (g)^*^	1.696 (296.61)	1.864 (272.77)	0.11
Gestational age at birth (weeks)^**^	33 (32–35)	34 (32–35)	0.80
Apgar 1 min^**^	8 (8–9)	8 (7–8)	0.31
Apgar 5 min^**^	9 (8–9)	9 (8–9)	0.86
Hospital stay (days)^*^	26 (13)	24 (10)	0.55
Neonatal medical index (*n*/%)			
*Level 1 and 2*	9 (56.30)	11 (73.30)	
*Level 3*	7 (43.80)	3 (20.00)	
*Level 4*	-	1 (6.70)	
Gender (*n*/%) ^∞^			0.07
*Male*	6 (37.50)	11 (73.30)	
*Female*	10 (62.50)	4 (26.70)	
Chronological age at baseline (weeks)^**^	36 (35–41)	36 (35–39)	0.66
Chronological age at 2 months assessment (weeks)^**^	14 (12–16)	15 (13–16)	0.14
Chronological age at 3 months assessment (weeks)^**^	17 (16–20)	18 (18–22)	0.05
Chronological age at 4 months assessment (weeks)^**^	20 (19–24)	24 (21–24)	0.02

* Values presented as mean (standard deviation)—T Student Test.

** Values presented as median (interquartile interval 25–75)—Mann–Whitney U Test.

∞ Fisher Exact Test.

The gestational age of the preemies, but not the age at hospital discharge, was controlled according to the inclusion criteria: caregivers with preemies born between 30 and 36 weeks and six days confirmed by ultrasound, adequate gestational age with a normal or poor repertoire in the general movements assessment. Exclusion criteria included brain injuries (e.g., intraventricular hemorrhage [grade 3 or 4], periventricular white matter injury, or hypoxic–ischemic encephalopathy), genetic syndrome (e.g., trisomy 21), or musculoskeletal deformity (e.g., limb deficiency). Medical complications and the neonatal medical index (ranges from 0 to 5; high scores indicate a higher risk of comorbidities) were extracted from the medical records of the preemies (Korner et al., 1993). After randomization, preemies who experienced any decline in health requiring hospitalization, who were relocated out of the state, or whose caregivers did not answer the contact were also excluded.

### Retention and attrition

**Figure 1:**
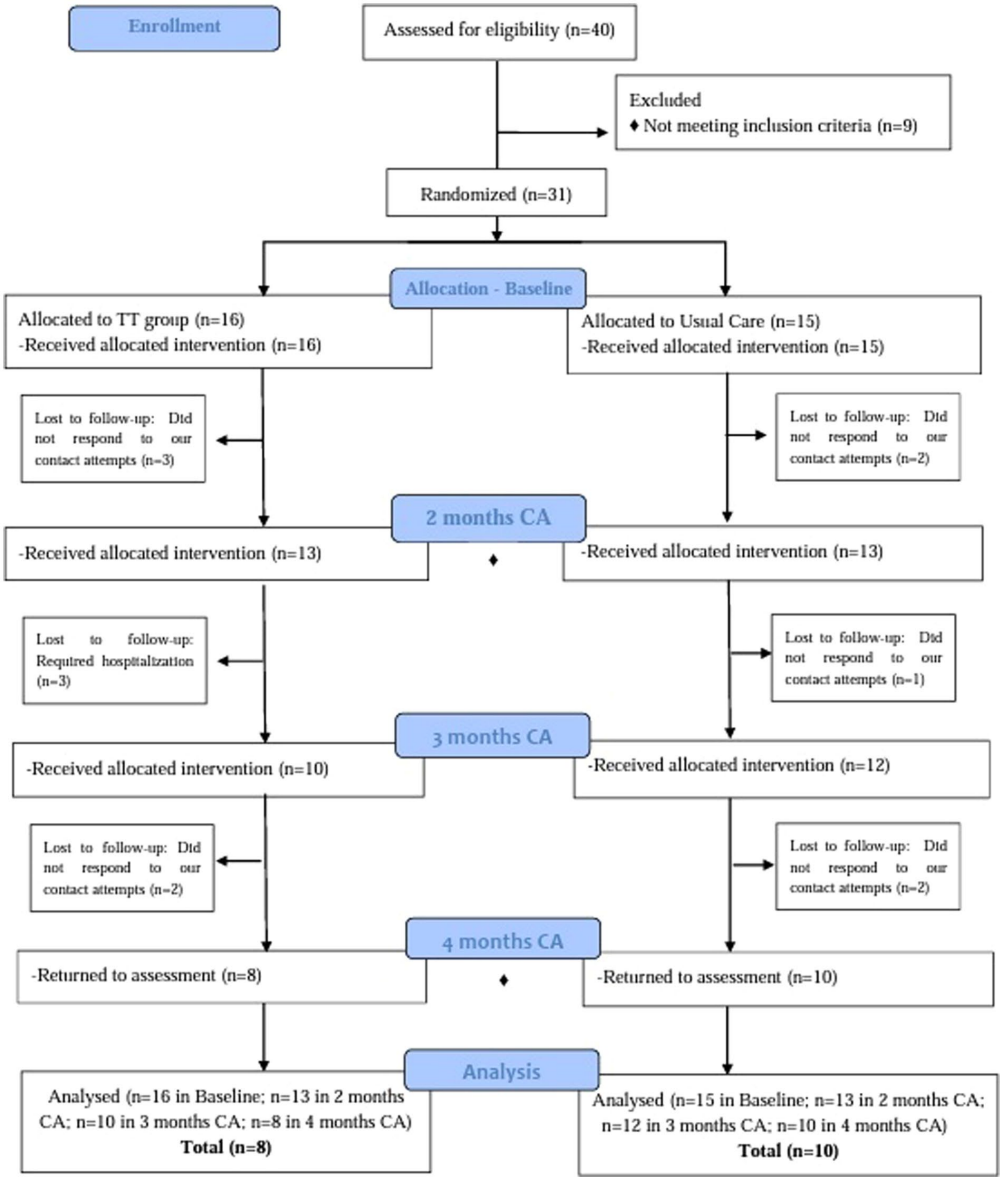
CONSORT flow chart. This flow chart showing the recruitment and retention of participants in each arm of the clinical trial. The kinematic analysis in the Kinovea® software was used in all assessments. ♦ Scale Bayley was administered at 2 and 4 months of corrected age.

Forty preemies were eligible among the 60 enrolled during the study period; 31 provided their informed consent to participate in the study and completed the baseline assessment. Afterward, 26 and 18 preemies completed the assessment after two months and four months of corrected age, respectively (eight from the TT group and 10 from the usual care [UC] group). Three preemies from the TT group required hospitalization, and 10 caregivers (five from each group) did not reply to the contact attempts. Figure 1 illustrates the recruitment and retention of participants.

### Randomization and blinding

Preemies were randomized to TT or UC groups after baseline assessment using a computer-generated randomization sequence (Dallal, 2013). Both groups received UC as it would be unethical to withhold routine care. UC comprised the kangaroo mother care (KMC) (Brazil, 2017), a well-established practice known for its proven short- and mid-term effectiveness and safety that engages parents as the primary caregivers to meet the biological needs of the preemie (Dhage et al., 2023).

The KMC was implemented in Brazil by the Ministry of Health to guide the care of low-birth-weight preemies as part of policies for humanizing maternal and child care (Brazil, 2017). During hospitalization, KMC constitutes UC according to two components: (1) the kangaroo position that provides continuous skin-to-skin contact between mother and infant, resulting in thermal regulation and other benefits, and (2) exclusive breastfeeding. After hospital discharge, as part of the ambulatory care routine, physicians also verify the nutritional status based on weight, height, and body mass index-for-age and refer preemies to specialized services if needed (e.g., physiotherapy, psychology, nutritionist, phono audiology, and others). Furthermore, parents and caregivers were encouraged to increase the family interaction with the preemies using toys and active verbal communication (Brazil, 2017).

**Figure 2:**
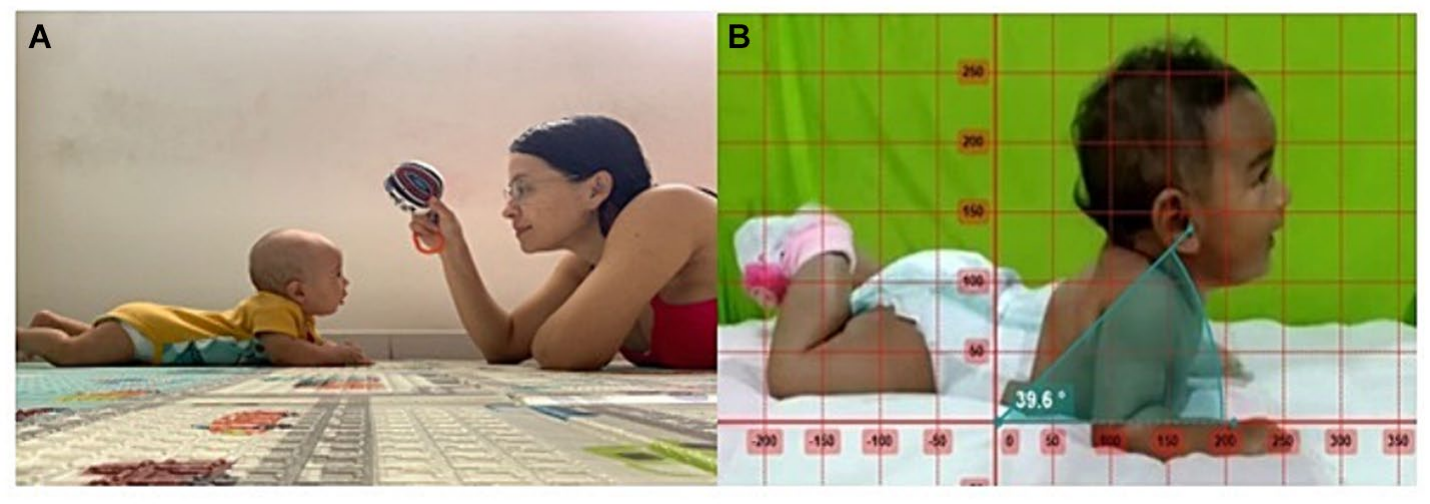
**(A)** Tummy time practice carried out by mother according to the handout. **(B)** The kinematic analysis in the Kinovea® software was performed using a 2D.

Caregivers from both groups received orientation about each protocol. Parents from the TT group were asked to place the preemies in the prone position for at least 20 min during daily life and playtime. During TT, parents were encouraged to stimulate their preemies using toys, sit or lie in front of them, maintain eye contact, engage in verbal communication, or sing (Figure 2A); illustrative examples for the TT were provided in a booklet (available in Supplementary material). The parents received these instructions during the first ambulatory care consultation on the same day they were invited to participate in the study.

Telemonitoring visits were conducted at least once a week for all parents until their preemies reached three months of corrected age. During these visits, the interventionist reinforced the UC guidelines for all parents and provided additional guidance for TT. Parents were encouraged to register in a booklet the number of minutes the preemie remained in the prone position each day to ensure adherence to TT (available in the Supplementary material). The study interventionist (SSSS) was a board-certified pediatric physical therapist with experience in providing intervention in neonatal care and the first months of life. Moreover, SSSS received training and attended regular meetings to discuss intervention strategies with the researchers SAP and CDLA.

All assessments were blinded. Two researchers not involved in the study (NAAR and TLGS) conducted the assessments, and data were tabulated using predetermined encoding by another blinded researcher (IAG). The results were not disclosed to the study interventionist, and parents were instructed not to reveal the intervention they were performing.

### Outcomes and follow-up

All preemies were assessed in person on the same schedule by a researcher blinded to the group assignment. However, the PHE assessment after three months of corrected age was recorded by the parents and sent via WhatsApp® to the interventionist. This evaluation was online to ensure the continuity of assessments even in financial and transportation difficulties. The PHE was assessed at baseline, two months (eight weeks after baseline), three months (end of follow-up), and four months (one month after the end of follow-up) of corrected age. Motor and cognitive development were assessed after two and four months of corrected age since they needed to be performed in person.

### Primary outcomes

#### Prone head elevation (PHE)

PHE was measured using the Kinovea® software version 0.9.5 (Joan Charmant & Contributors, Bordeaux, France). The kinematic analysis was performed using a 2D measurement of movements recorded during the prone position, which considered the angle from the tragus of the ear to the supporting surface of the preemie (Bentzley et al., 2015; Figure 2B). This software presents adequate reliability, validity, and reproducibility for analyzing dynamic movements (Fernández-González et al., 2020; Puig-divı et al., 2019). All assessments were performed individually in the presence of caregivers. The PHE was recorded for 2 min for all preemies. During the recording, they were encouraged to focus on a toy displayed between 4 and 6 in. above the eye line.

#### Secondary outcome measures

#### Motor and cognitive development

Bayley Scales of Infant and Toddler Development, third edition (Bayley-III) was used to assess the motor and cognitive development at two and four months of corrected age. This scale was translated, validated, and adapted for Brazilian children (Madaschi et al., 2016). Normative values on the Bayley-III include composite scores for cognitive and motor outcomes with a mean of 100 and a standard deviation of 15. All assessments were performed individually in the presence of caregivers at the pediatrics and childcare ambulatory care. An interventionist trained in the Bayley-III scale conducted all the assessments following the guidelines (Bayley, 2006).

At two and four months, the motor assessment using the Bayley-III primarily focused on the head control in the vertical, ventral suspension, and prone position, as well as their duration. On the other hand, the cognitive assessment encompassed items related to the interaction of the baby with the environment, such as object observation, exploration, and visual preference (Bayley, 2006).

The reversal rule was considered in the assessments, as the infant scored 1 on the first three items from the starting point compatible with their age. The age-corrected score was considered in the case of a preemie. However, the assessment returned to the corresponding starting point for the previous age if the preemie scored zero in any of the first three items, and was interrupted if the preemie scored zero in five consecutive items (Bayley, 2006).

### Statistical analysis

The *a priori* statistical analysis considered that data collection from 14 preemies (seven per group) provided the statistical power needed to detect differences between groups in the primary outcomes with an alpha of 5 and 80% power (29). Data normality was assessed using the Shapiro–Wilk test. Bayley motor composite and cognitive composite scores were expressed as mean and standard deviation.

Frequency distribution and percentages described categorical variables. The Mann–Whitney U test was used to compare medians (number of infants, Apgar, and chronological age). The chi-square test or Fisher exact test (for the categories with less than five cases) was applied for gender, marital status, educational level, familiar income, and housing between TT and UC groups.

PHE was assessed using the linear mixed model according to the group (TT and UC) and time (i.e., baseline, two months, three months, and four months).

Bayley-III composite scores were compared using the Student’s t-test for unpaired samples from both groups at two and four months of corrected age. Additionally, effect sizes were calculated to verify the differences between the Bayley-III composite scores (motor and cognitive components) after two and four months of corrected age. The significance level adopted was 5%. All analyses were conducted using the Statistical Package for Social Science (SPSS) software, version 25 (IBM Corp., USA).

## Results

All families were from the Brazilian northeast, self-declared brown, completed prenatal care, and presented poor socioeconomic conditions. Most mothers (77.4%) reported no household income, and seven (22.6%) lived in rural areas. Among the 31 preemies, 10 were delivered via cesarean section, and three required mechanical ventilation during hospitalization. None of the preemies received sedation medication, and the hospital length of stay ranged from 8 to 54 days. Based on the neonatal medical index, 20 preemies were classified at level 1 or 2, 10 at level 3, 1 at level 4, and no preemie ranked at level 5 (Table 1).

### Description of UC group

All caregivers performed KMC daily at home and maintained exclusive breastfeeding. The nutritional status percentiles for all preemies were between the 5th and 95th curves (WHO Multicentre Growth Reference Study Group, 2006), and none were referred to specialized infant rehabilitation services. The mean duration of telemonitoring visits ranged from five to 15 min (mean of 9 min) twice a week, and the intervals between meetings ranged from two to three days. Caregivers usually presented no doubts about the routine care.

### Description of TT group

The mean time for TT was 17 min, ranging from 10 to 30 min per day. All caregivers self-declared enjoying having playtime with their preemies during TT. The mean duration of telemonitoring meetings ranged from 10 to 20 min (mean of 13 ± 3 min) twice a week with intervals ranging from two to three days. Caregivers usually presented no doubts regarding the positions illustrated in the booklet. Moreover, three preemies from the TT group were hospitalized with respiratory infections.

### Primary outcomes

#### Prone head elevation (PHE)

**Figure 3:**
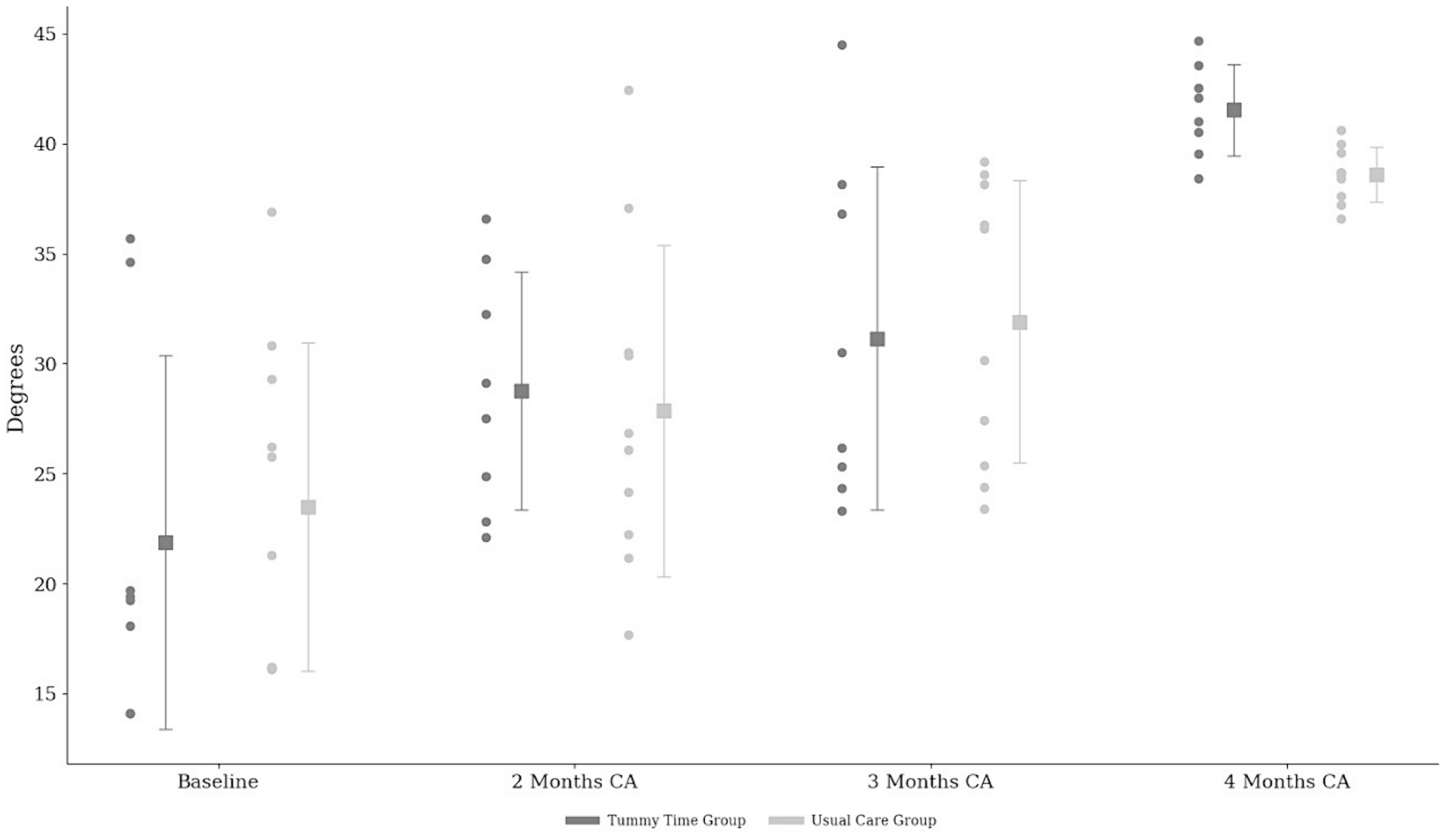
The angles for prone head elevation between groups over a timeline and the individual trajectories of each subject. Values presented as mean and the individual trajectories of each subject throughout the assessments. ▪ Mean values for each group and time period and the standard deviation; CA, corrected age.

PHE was 23.59 ± 7.67 degrees in both groups at baseline and increased up to 39.77 ± 2.73 degrees at four months of correct age. The comparison of the degrees between groups over the timeline is shown in Figure 3. The PHE presented significant interaction between time and the increase in the PHE angle (*p* = 0.001). However, no significant group-time interactions were observed between groups (*p* = 0.668; Table 2). The calculated effect sizes are shown in Table 2. The PHE degrees increased to 4.77 degrees after two months (*d* = 1.58), 9.14 degrees after three months (effect size = 1.10), and 16.48 degrees after four months (effect size = 0.61) compared with baseline (evaluation 1) (Table 2).

**Table 2:** Linear mixed model to cervical control.

Parameters	Estimative	Effect size (*d*)	Degrees of freedom	*p*-value
Intercept	39.61	*	62.46	<0.001
Time 1—Baseline	−16.48	1.58	46.32	<0.001
Time 2—2 months	−11.71	1.10	46.12	<0.001
Time 3—3 months	−7.34	0.61	47.53	0.036
Time 4—4 months	0	*		*
Tummy time	0.94	*	24.46	0.668
Usual care group	0	*		

*Data non-available for usual care group X tummy time. Effect sizes calculated only for Byley-III.

### Secondary outcomes

#### Developmental outcomes

**Figure 4:**
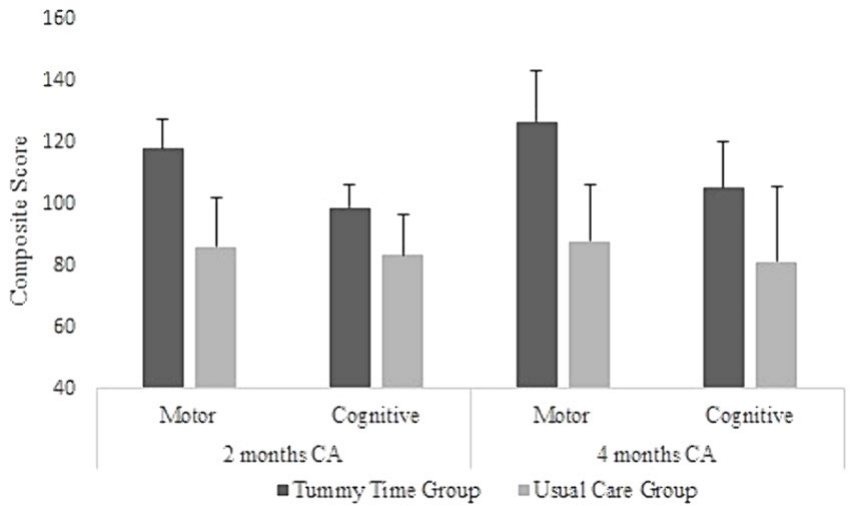
Bayley composite scores for motor and cognitive components and groups. Values presented as mean and the standard deviation; CA, corrected age.

During the study period (October 2021 to September 2022), 26 Bayley-III assessments were administered at two months, and 18 after four months of corrected age. The Bayley-III motor composite score was significantly different between TT (117.85 ± 9.67) and UC (85.91 ± 13.81) groups after two months (*t* [6.71] = 19.27, *p* < 0.001), with effect size *d* = 2.81. This component was also different between TT (126.62 ± 16.15) and UC (87.80 ± 18.54) groups after four months of corrected age (*t* [4.74] = 15.84, *p* < 0.001); effect size of *d* = 1.46 (Figure 4).

The Bayley-III cognitive composite score of the TT group was increased (98.57 ± 7.44) compared with the UC (83.33 ± 13.37) group after two months (*t* [3.50] = 16.63, *p* = 0.003), presenting an effect size of *d* = 2.30. Importantly, two preemies from the UC group presented results below the mean. Furthermore, the TT group presented an increased cognitive composite score (105.00 ± 14.88) compared with the UC group (81.00 ± 24.24) after four months of corrected age (*t* [2.58] = 15.15, *p* = 0.021); effect size of *d* = 1.20 (Figure 4). The UC group presented scores that were one standard deviation below the mean on the composite cognitive scale at both ages assessed. All preemies scoring below one standard deviation on the composite score of the Bayley Scale in both motor and cognitive skills were referred to specialized infant rehabilitation services.

### Participants who dropped out

Thirty-one preemies were assessed at baseline, and 18 were reassessed after four months of corrected age. Sixteen preemies were included in the TT group: two withdrew at two months of corrected age and six at three months. Fifteen preemies were included in the UC group: three withdrew at two months, two at three months, and two at four months of corrected age. Among those who dropped out, two (15.4%) lived in rural areas. The distance between the residences to the pediatrics and childcare ambulatory ranged from 12.42 to 152.85 miles. Table 3 describes the socioeconomic conditions of the families of the remaining and dropped preemies. No significant differences were observed between groups.

**Table 3:** Description of retention and no retention of families in the intervention.

	Retention (*n* = 18)	No retention (*n* = 13)	*p*-value
Maternal age^*^	29 (6.90)	29 (7.71)	0.92
Number of children at home^**^	2 (1–3)	2 (1–4)	0.70
Marital status (*n*/%)^∞^			1.00
*Single*	2 (11.10)	4 (30.80)	0.20
*Married*	16 (88.90)	9 (69.20)	
Maternal education (*n*/%)^∞^			0.06
*Elementary or less*	4 (22.20)	8 (61.50)	
*High school or more*	14 (77.80)	5 (38.50)	
Employment (*n*/%)^∞^			0.19
*Yes*	6 (33.30)	1 (7.70)	
*No*	12 (66.70)	12 (92.30)	
Residence (*n*/%)^∞^			0.66
*Metropolitan area*	13 (72.20)	11 (84.60)	
*Countryside*	5 (27.80)	2 (15.40)	
Distance (*n*/%)^∞^^,^ ^***^			0.48
47*.73 miles or less*	11 (61.10)	6 (46.20)	
*47.73 miles or more*	7 (38.90)	7 (53.80)	

* Values presented as mean (standard deviation)—T Student Test.

** Values presented as median (interquartile interval 25–75)—Mann–Whitney U Test.

∞ Fisher Exact Test.

*** Values presented as mean of distance between the distance of the cities to ambulatory care.

## Discussion

This randomized clinical trial was the first study to assess the effect of daily TT on the PHE and motor and cognitive development of preemies. Considering that previous studies discussed how different stimuli in environmental exploration trigger a developmental cascade that improves motor and cognitive development (Adolph and Hoch, 2019; Kretch et al., 2014; Lee and Galloway, 2012; Soska and Adolph, 2014), this study hypothesized whether daily TT could improve motor and cognitive development in preemies compared with those not engaged in this practice.

The present study showed that TT may increase PHE degree over time, confirming the first hypothesis. This position contributes to early development by strengthening head, neck, and trunk muscles, enhancing mobility and motor control (Adolph and Hoch, 2019; Lee and Galloway, 2012; Wentz, 2017).

The preemies from the TT group presented improved motor and cognitive scores compared with those from the UC group. Furthermore, these findings were associated with enhanced PHE angle, corroborating the second hypothesis of this study. The TT stimulates preemies to develop motor, cognitive, and sensory skills by presenting new environmental challenges. Moreover, TT enhances manual dexterity and visual function, improving accuracy and attention. These physical and sensory experiences contribute to varied developmental paths, enhancing problem-solving skills and cognitive abilities in infants (Adolph and Hoch, 2019; Hadders-Algra, 2018; Kretch et al., 2014).

A longitudinal study addressing the benefits of TT on motor and cognitive outcomes showed that healthy full-term infants presented improved motor development between one and six months of life (Silva et al., 2023). Therefore, infants who practiced the prone position were more likely to achieve motor skills expected for their age. Furthermore, a systematic review showed that daily parent-delivered interventions were more effective in improving cognitive and motor outcomes in preemies than other interventions in the short and possibly long term (Khurana et al., 2020), corroborating the positive association between TT and improved cognitive performance observed in the present study.

Although the PHE angle increased over time, its values were not different between groups. This finding is possibly related to the UC associated with the KMC performed by both groups (Brazil, 2017). This method involves positioning infants in a kangaroo position on the chest of the parent to maximize skin-to-skin contact. This positioning supports physiological benefits, such as temperature regulation, bonding, and breastfeeding (Akbari et al., 2018; Dhage et al., 2023; Durmaz et al., 2023), and also facilitates neck muscle activation and head training (Wang et al., 2021). Since isolating the effects of the two interventions is unfeasible as it would be unethical, further studies could assess the existence of a cumulative effect.

The effects of TT may also be extended to preemies at risk of developmental delays due to immature neurological (Altimier and Phillips, 2018) or musculoskeletal (Drzał-grabie, 2019) systems. Although TT is already recommended for preemies (Clark and Kingsley, 2020), previous studies did not investigate its effects in this population. Therefore, encouraging TT may be a supportive strategy for parents of preemies to enhance their movements. Active involvement and empowerment of families are crucial factors in improving the environment and the development of preemies (Ferreira et al., 2020).

Early stimulation by family members promotes daily stimuli that improve the development of preemies, as well as the development of knowledge, capacity, and ability of the parents to identify the needs of their preemies (Ferreira et al., 2020). The best motor and cognitive results associated with early stimulation are related to the environmental setup that supports the self-regulatory mechanisms of the preemie and opportunities for exploration, promoting the development of primary and secondary variability. All these aspects are facilitated by direct interaction with their parents (Hadders-Algra, 2018). In addition, this type of intervention provides opportunities for early stimulation in low-income countries, where part of the population faces socioeconomic barriers and difficult access to specialized health centers (Nanyunja et al., 2022). The TT promotes family participation in a cost-effective intervention starting after hospital discharge, empowering the care of parents for their preemies.

Although this study stands as the first clinical trial examining the impacts of TT in preemies, the interpretation of the results may be limited due to the number of dropouts during the study. Notably, the Northeast region of Brazil ranks among the areas with the highest levels of vulnerability to poverty. This circumstance often leads to financial constraints to access transportation and specialized healthcare facilities (Brazil, 2024).

In this sense, strategies must be implemented to ensure access to interventions that reduce the risk of developmental delays in vulnerable infants, including preemies. Therefore, the TT is a feasible strategy to facilitate early intervention in low- and middle-income countries by empowering parents to establish a daily routine with their preemies. This intervention may be encouraged during hospitalization and reinforced during outpatient follow-up. Thus, healthcare professionals must be stimulated to disclose information using educational approaches (e.g., illustrative booklets).

## Conclusion

The PHE increased over time but it was not significantly different between groups. Nevertheless, the results suggest that the TT possibly improves the motor and cognitive development of preemies. This finding may be a feasible strategy in early stimulation to reduce barriers to access in low- and middle-income countries.

## Data Availability

The raw data supporting the conclusions of this article will be made available by the authors, without undue reservation.
