# Supplementary material for: Cognitive and motor improvement by tummy time practice in preemies from low-income settings: a randomized clinical trial
Source: Front Psychol. 2024 Sep 18;15:1289446. doi: 10.3389/fpsyg.2024.1289446 (PMC11445822; doi:10.3389/fpsyg.2024.1289446)
Supplement: Supplementary file 1 [file Data_Sheet_1.PDF]

WHAT IS TUMMY TIME?

It's the moment of the day that the baby plays laying on their stomachs when...

- Awake
- Under supervision

Start with short periods and increase with time!

Start with short periods of 2 - 3 minutes until you complete 20 minutes daily of Tummy Time.

Respect your baby's limits by recognizing their signs of tiredness:

- Crying
- Resting head on surface

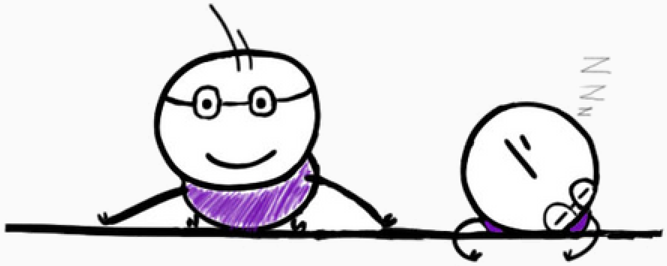

You can stop Tummy Time even before your baby gets tired

CONTACT US!

Department of Physical Therapy - Federal University of Rio Grande do Norte (UFRN)

gpsafeufrn@gmail.com

@gpsafeufrn

TUMMY TIME

How to do it with my baby?

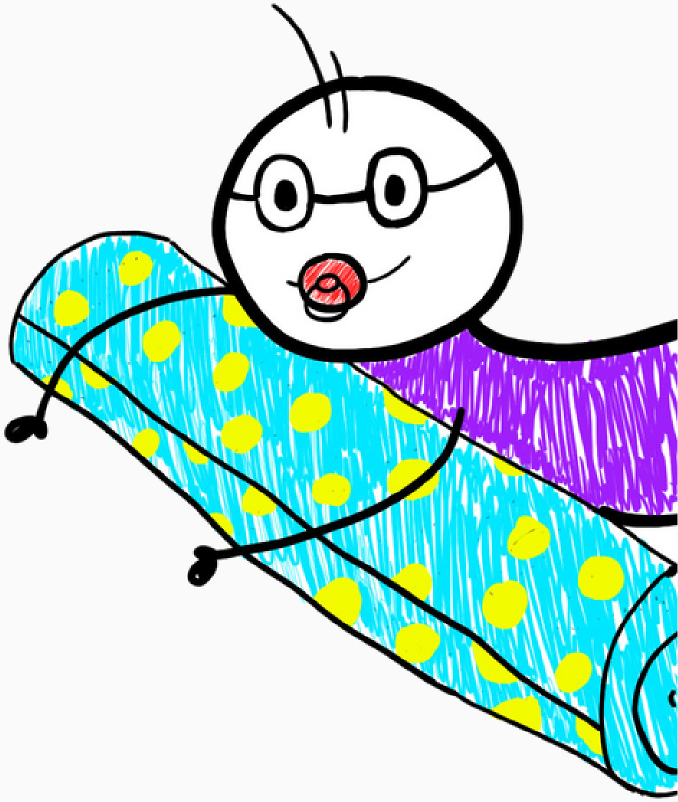

UFRN UNIVERSIDADE FEDERAL DO RIO GRANDE DO NORTE

WHY SHOULD I DO IT?

- It helps strengthen head, neck and upper boddy muscles
- Improves motor development and motor skills
- Prevents Plagiocephaly (flat spots of the back of the baby's head)

MAKE IT A DAILY ROUTINE

Insert Tummy Time into your usual daily activities:

- When drying the baby after shower
- When changing diapers or applying lotion
- Play time

After burping your baby, try placing him belly-down on your chest.

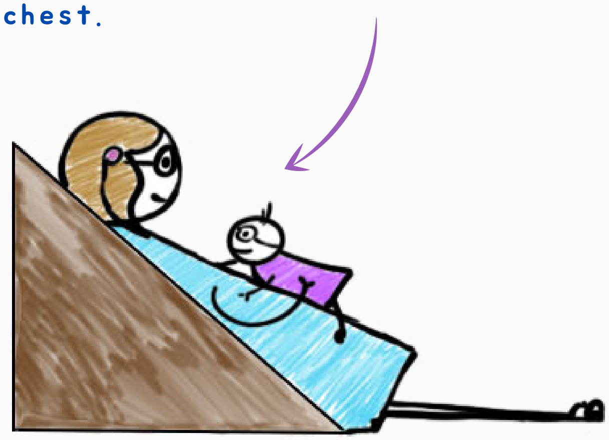

STIMULATE THE BABY

Stimulate your baby's ability to reach and play:

- Arrange toys in a circle around your baby, give preference to colorful toys that make sounds and arouse interest like a mirror

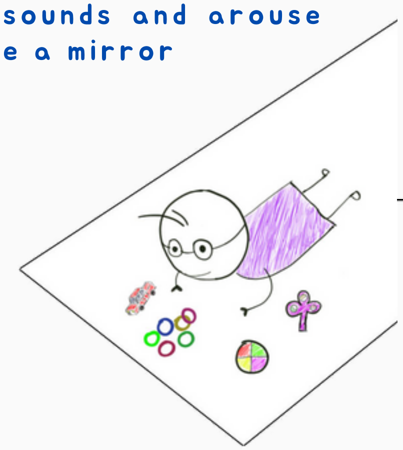

- Sit or lie down in front of the baby
- Hold a toy in front of the baby's face to get his attention

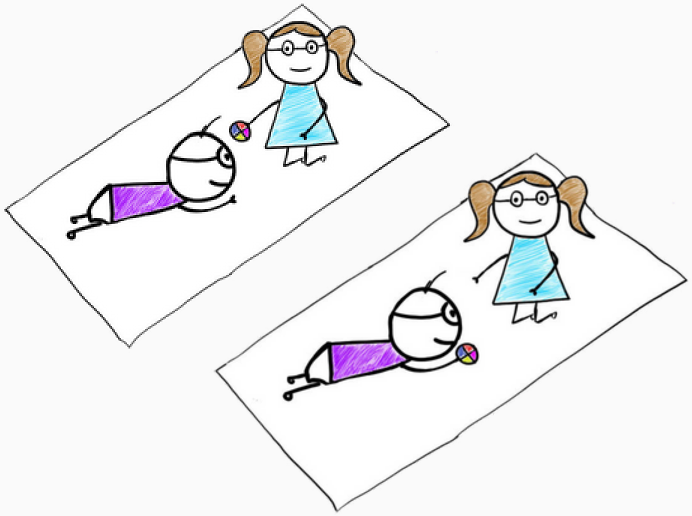

IMPROVE THE BOND

- Invite your family to join Tummy Time
- Make eye contact, speak or sing ,

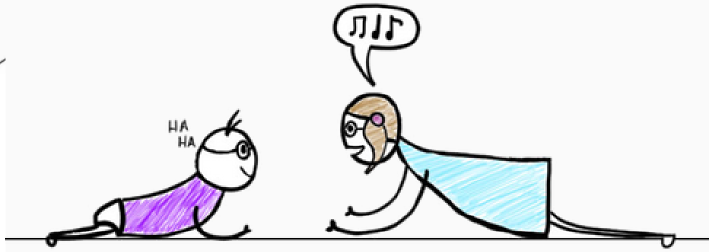

TIPS

Chest support

- Roll a thin towel or blanket and place it under your baby's chest and place his arms over the roll with his hands outstretched in front

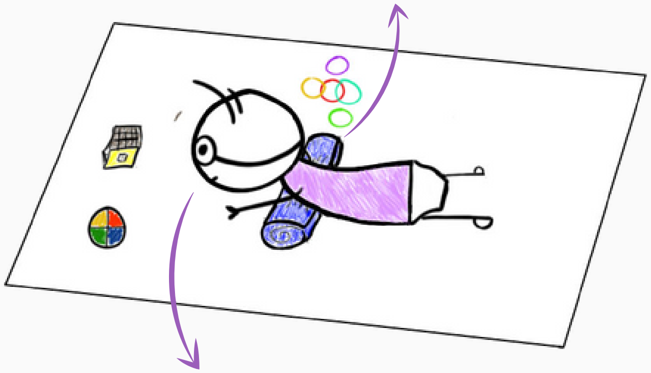

Your baby's chin should always be positioned in front of the chest support so that the nose and mouth are free

Tummy Time Log

week nº: ____________

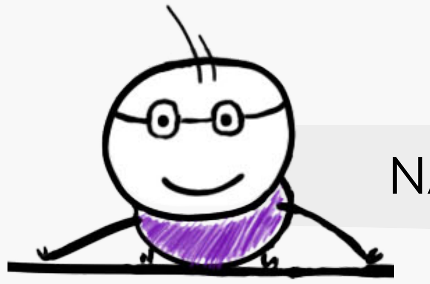

NAME:

	DAY 1	DAY 2	DAY 3	DAY 4	DAY 5	DAY 6	DAY 7
WEEK 1							
WEEK 2							
WEEK 3							
WEEK 4							
WEEK 5							

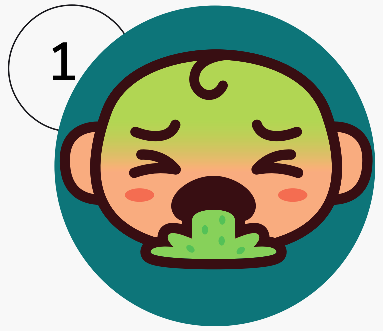

VOMIT

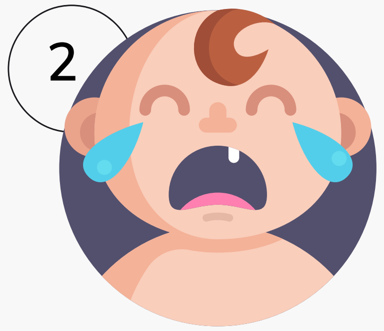

CRYING

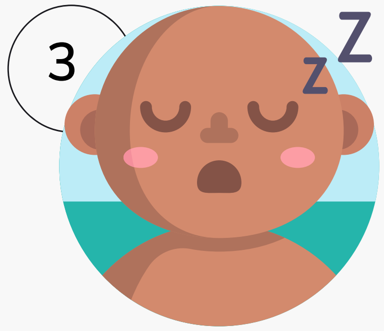

ASLEEP

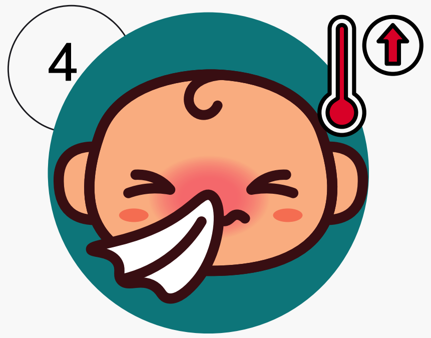

FEVER
